# MALT1 regulates Th2 and Th17 differentiation *via* NF-κB and JNK pathways, as well as correlates with disease activity and treatment outcome in rheumatoid arthritis

**DOI:** 10.3389/fimmu.2022.913830

**Published:** 2022-07-28

**Authors:** Qiubo Wang, Yapeng Wang, Qingyang Liu, Ying Chu, Rui Mi, Fengying Jiang, Jingjing Zhao, Kelong Hu, Ran Luo, Yufeng Feng, Harrison Lee, Dong Zhou, Jingyi Mi, Ruoyu Deng

**Affiliations:** ^1^ Department of Clinical Laboratory, Wuxi 9th People's Hospital Affiliated to Soochow University, Wuxi, China; ^2^ Department of Orthopedics, Wuxi 9th People's Hospital Affiliated to Soochow University, Wuxi, China; ^3^ Department of Laboratory and Statistics, Shanghai QeeJen Bio-tech Institution, Shanghai, China; ^4^ Department of Research Design, Shanghai QeeJen Bio-tech Institution, Shanghai, China; ^5^ Department of Bioengineering, Chonnam National University, Gwangju, South Korea; ^6^ Department of Rheumatology, The Royal Melbourne Hospital, Melbourne, VIC, Australia; ^7^ Department of Sport Medicine, Wuxi 9th People's Hospital Affiliated to Soochow University, Wuxi, China; ^8^ Department of Life Science, The Fudan University, Shanghai, China

**Keywords:** MALT1, rheumatoid arthritis, Th1/2/17 cells, treatment outcome, NF-κB and JNK pathways

## Abstract

**Objective:**

MALT1 regulates immunity and inflammation in multiple ways, while its role in rheumatoid arthritis (RA) is obscure. This study aimed to investigate the relationship of MALT1 with disease features, treatment outcome, as well as its effect on Th1/2/17 cell differentiation and underlying molecule mechanism in RA.

**Methods:**

Totally 147 RA patients were enrolled. Then their blood Th1, Th2, and Th17 cells were detected by flow cytometry. Besides, PBMC MALT1 expression was detected before treatment (baseline), at week (W) 6, W12, and W24. PBMC MALT1 in 30 osteoarthritis patients and 30 health controls were also detected. Then, blood CD4^+^ T cells were isolated from RA patients, followed by MALT1 overexpression or knockdown lentivirus transfection and Th1/2/17 polarization assay. In addition, IMD 0354 (NF-κB antagonist) and SP600125 (JNK antagonist) were also added to treat CD4^+^ T cells.

**Results:**

MALT1 was increased in RA patients compared to osteoarthritis patients and healthy controls. Meanwhile, MALT1 positively related to CRP, ESR, DAS28 score, Th17 cells, negatively linked with Th2 cells, but did not link with other features or Th1 cells in RA patients. Notably, MALT1 decreased longitudinally during treatment, whose decrement correlated with RA treatment outcome (treatment response, low disease activity, or disease remission). In addition, MALT1 overexpression promoted Th17 differentiation, inhibited Th2 differentiation, less affected Th1 differentiation, activated NF-κB and JNK pathways in RA CD4^+^ T cells; while MALT1 knockdown exhibited the opposite effect. Besides, IMD 0354 and SP600125 addition attenuated MALT1’s effect on Th2 and Th17 differentiation.

**Conclusion:**

MALT1 regulates Th2 and Th17 differentiation *via* NF-κB and JNK pathways, as well as correlates with disease activity and treatment outcome in RA.

## Introduction

Rheumatoid arthritis (RA) is a common autoimmune and inflammatory disease featured by synovium hyperproliferation and related synovitis, which affects nearly 1% population worldwide (1, 2). Apart from the direct joint swelling and bone erosion, RA patients also suffer from extraarticular manifestations and comorbidities involving dermatologic, ophthalmologic, pulmonary, cardiovascular, neurologic, and hematologic organ systems (3–5). To improve RA outcomes, the efforts exploring RA underlying pathogenesis are always on the way and never stop, through which the corresponding treatments are proposed. Although the detailed pathogenesis of RA is still unclear, genetic factors, aberrant autoimmune antibodies (rheumatoid factor (RF), anti-cyclic citrullinated peptide antibody (ACPA), etc.), dysregulated immune cells (such as B cells, CD4^+^ T cells, etc.), and excessive systemic inflammation contribute to RA development and progression (6–8).

Mucosa-associated lymphoid tissue lymphoma translocation protein 1 (MALT1), a multi-functional factor transmitting signals of immune receptors with an immunoreceptor tyrosine-based activation motif (ITAM) to activate the NF-κB pathway to regulate a large number of immune cell functions, including T cells, B cells, NK cells, dendritic cells and macrophages (9–11). Meanwhile, MALT1 facilitates exacerbated inflammation *via* modifying NF-κB and MAPK pathways (12). On account of the above-mentioned functions, MALT1 is observed to be closely involved in the pathogenesis of immune and inflammatory diseases such as psoriasis, inflammatory bowel disease (IBD), Sjogren’s syndrome, ankylosing spondylitis, etc. (13–16). In the aspect of RA, MALT1 not only may participate in its pathology *via* regulating immune cells and inflammation (9–12), but also promotes RA development *via* a T-cell signaling dependent way and NF-κB signaling activation (17–19). Furthermore, MALT1 relates to CD4^+^ T cell [T helper (Th) cell] subpopulations and is dysregulated in RA (20, 21). However, the implication of MALT1 in RA management is still largely unknown.

This study aimed to investigate the relationship of MALT1 with disease features, treatment outcome, as well as its effect on Th1/2/17 cell differentiation and underlying molecule mechanism in RA.

## Methods

### Participants

This study serially enrolled a total of 147 active RA patients treated between March 2018 and February 2021. The enrollment criteria were set as (a) diagnosed as RA according to the 2010 RA classification criteria issued by American College of Rheumatology (22); (b) aged over 18 years; (c) had a disease activity score of 28-erythrocyte sedimentation rate (DAS28_ESR_) more than 3.2; (d) volunteered to participate in the study. Patients who had the following conditions were ineligible for enrollment: (a) presented as active infections; (b) had moderate to severe dysfunction in liver or kidney; (c) had a history of hematologic malignancy or cancer; (d) had severe joint deformity; (e) female patients during pregnant or lactating. Additionally, this study also recruited 30 osteoarthritis (OA) patients as disease controls and 30 healthy subjects as healthy controls (HCs). The age and gender of OA patients and HCs were matched to RA patients. 30 reactive arthritis patients with age and gender matched to RA patients were also enrolled as another kind of disease controls. The study was permitted by Ethics Committee of Wuxi 9th People's Hospital Affiliated to Soochow University. All participants signed the informed consent.

### Collection of clinical characteristics and samples

Clinical characteristics of RA patients were documented after recruitment for study use. Peripheral blood (PB) samples were collected from RA patients before treatment initiation (W0, N=147), as well as from OA patients (N=30), HCs (N=30) and reactive arthritis patients after enrollment. Besides, PB samples were also collected from RA patients at 6 weeks after treatment initiation (W6, n=144), at 12 weeks after treatment initiation (W12, n=137), and at 24 weeks after treatment initiation (W24, n=119).

### Detection of clinical samples

After PB sample collection, peripheral blood mononuclear cell (PBMC) samples were isolated using Ficoll (Cytiva, USA) to detect MALT1 expression by reverse transcription-quantitative polymerase chain reaction (RT-qPCR). Besides, the fresh PB samples were used to evaluate the proportions of Th1 cells, Th2 cells, and Th17 cells in CD4^+^ T cells by flow cytometry.

### Treatment for RA patients

RA patients received treatment for 24 weeks, and the treatment regimens were prescribed according to actual disease status, generally including the conventional synthetic disease-modifying antirheumatic drugs (csDMARDs) combination, and biological disease-modifying antirheumatic drugs (bDMARDs) with or without csDMARDs.

### Clinical assessment for RA patients

Clinical response, clinical low disease activity (LDA), and clinical remission were assessed during a clinic-visit follow-up at W6, W12, and W24 according to the clinical assessment criteria for RA patients (23, 24). Clinical response was defined as a decline of DAS28_ESR_ > 1.2; clinical LDA was defined as DAS28_ESR_ ≤3.2; clinical remission was defined as DAS28_ESR_ ≤2.6. Clinical response, clinical LDA, and clinical remission at W24 were respectively used to classify RA patients as response and non-response patients; LDA patients and non-LDA patients; remission and non-remission patients. The RA patients who were lost to follow-up or withdrew from the study early for poor efficacy or adverse events were included in the analysis based on the intention-to-treat (ITT) principle, and the missing data were measured by the last observation carried forward (LOCF) method.

In addition, clinical disease activity index (CDAI) was also evaluated, then clinical LDA and remission based on CDAI at W6, W12, and W24 was also assessed. LDA and remission by CDAI were respectively used to classify RA patients as LDA patients and non-LDA patients; remission and non-remission patients, as well.

### Naïve CD4^+^ T cell isolation and polarization

Naïve CD4^+^ T cells were separated from PBMC samples using naïve CD4^+^ T cell Isolation kit (Miltenyi, Germany) according to the kit’s protocol. The cells were activated with anti-CD28 (2 μg/mL, eBioscience, USA) and anti-CD3 (5 μg/mL, eBioscience, USA). The polarization of Th1/Th2/Th17 was performed for 3 days in the presence of different polarizing conditions (25, 26). For Th1 polarization, anti–IL-4 (10 μg/ml, eBioscience, USA) and IL-12 (10 ng/mL, Sigma, USA) were applied. For Th2 polarization, IL-4 (2.5 ng/mL, Sigma, USA) and anti–IFN-γ (10 μg/mL, eBioscience, USA) were applied. For Th17 polarization, TGF-β (5 ng/mL, Sigma, USA), IL-6 (10 ng/mL, Sigma, USA), IL-1β (10 ng/mL, Sigma, USA), IL-23 (20 ng/ml, Sigma, USA), anti–IFN-γ (10 μg/ml), and anti–IL-4 (10 μg/ml) were applied. Naïve CD4^+^ T cells were maintained in RPMI 1640 medium (HyClone, USA) supplemented with 10% FBS (HyClone, USA) at 37°C and 5% CO_2_ for all experiments. The expression of MALT1 in polarized cells was analyzed, and the naïve CD4^+^ T cells being cultured normally for 3 days were utilized as control.

### MALT1 regulation experiment

The MALT1 overexpression and knockdown lentivirus (anti-MALT1), as well as the scramble lentivirus, were obtained from Genepharma (Shanghai, China). For transfection, naïve CD4^+^ T cells were activated as mentioned for 48 h and afterward transfected with lentivirus using HilyMax (Dojindo, Japan) according to the manufacturer’s procedure. After being transfected for 48 h, cells were harvested for RT-qPCR and western blot. Meanwhile, transfected cells were resuspended in media supplemented with different polarizing conditions for Th1/Th2/Th17 polarization as aforementioned. After 3 days of incubation, supernatants were collected for enzyme-linked immunosorbent assay (ELISA) assays, and cells were harvested for flow cytometry assays.

### IMD 0354 (NF-κB antagonist) and SP600125 (JNK antagonist) treatment

Cells were seeded and transfected with MALT1 overexpression or scramble lentivirus as indicated. Then, IMD 0354 (NF-κB antagonist repressing NF-κB pathway) (0.2 μM, MCE, China) (27) and SP600125 (JNK antagonist repressing JNK pathway) (10 μM, MCE, China) (28) were added to validated the regulation of NF-κB and JNK signaling pathway by MALT1, respectively. Western blot assays were performed after 48 h treatment. Subsequently, Th2/Th17 polarization assays of transfected cells were performed as mentioned in the presence of IMD 0354 or SP600125. After 3 days of incubation, supernatants and cells were collected for ELISA assays and flow cytometry assays, respectively.

### ELISA and flow cytometry

ELISA kits obtained from Sangon Biotech (Shanghai, China) were applied for detecting the concentrations of IFN-γ, IL-4, and IL-17A in cell supernatants after Th1/Th2/Th17 polarization. For flow cytometry staining, PBMC samples or differentiated cells were treated with ionomycin (5 μM, Beyotime, China), phorbol myristate acetate (1 μM, Beyotime, China), and BD GolgiStop (1 μL/mL, BD, USA) for 4 h before collecting. Next, cells were exposed to fixation/permeabilization reagent (BD, USA) for 15 min and incubated with antibodies for another 30 min. Cells were then harvested and analyzed by a FACSCanto II (BD, USA). The antibodies were as follows: FITC anti–CD4 (BioLegend, USA), PE anti–IFN-γ (BD, USA), APC anti–IL-4 (MultiScience, China), and Alexa Fluor 647 anti–IL-17A (BD, USA).

### RT-qPCR

Total RNA from PBMC samples or naïve CD4^+^ T cells was extracted with Trizol (Invitrogen, USA). RT-qPCR was conducted using RT reagent Kit (Takara, Japan) and PCR Master Mix (Beyotime, China). The level of MALT1 was normalized to GAPDH based on the 2^-ΔΔCt^ method. The primer’s information was as follows: MALT1 forward: TCTTGGCTGGACAGTTTGTGA, MALT1 reverse: GCTC TCTGGGATGTCGCAA; GAPDH forward: GAGTCCACTGGCGTCTTCAC, GAPDH reverse: ATCTTGAGGCTGTTGTCATACTTCT.

### Western blot

Naïve CD4^+^ T cells were lysed using RIPA (Beyotime, China), and protein was quantified with the BCA kit (ThermoFisher, USA). Extracted proteins were separated using SDS-PAGE (Solarbio, China) and transferred onto NC membranes (Bio-rad, China). After being blocked by 5% BSA, membranes were further incubated with diluted primary antibodies and secondary antibodies for 1 h at 37°C, successively. Afterward, the bands were revealed using the ECL kit (ThermoFisher, USA). The antibodies information was shown in **Supplementary Table 1**.

### Statistics

Statistical analyses were performed using SPSS V.24.0 (IBM Corp., USA), and graphs were plotted using GraphPad Prism V.6.01 (GraphPad Software Inc., USA). For clinical data analysis, differences of variables among three groups were compared using the Kruskal-Wallis H rank-sum test, and differences between the two groups were analyzed using the Wilcoxon rank-sum test. Receiver operating characteristic (ROC) analysis was carried out to evaluate the ability of MALT1 in distinguishing different subjects. Correlations of two variables were determined using Spearman’s rank correlation test or Wilcoxon’s rank-sum test. Changes in MALT1 expression over time were evaluated using the Friedman test. For experimental analysis, comparisons among four groups were evaluated using one-way ANOVA with Tukey’s *post hoc* test. *P* value <0.05 was considered significant.

## Results

### Study flow presentation

**Figure 1:**
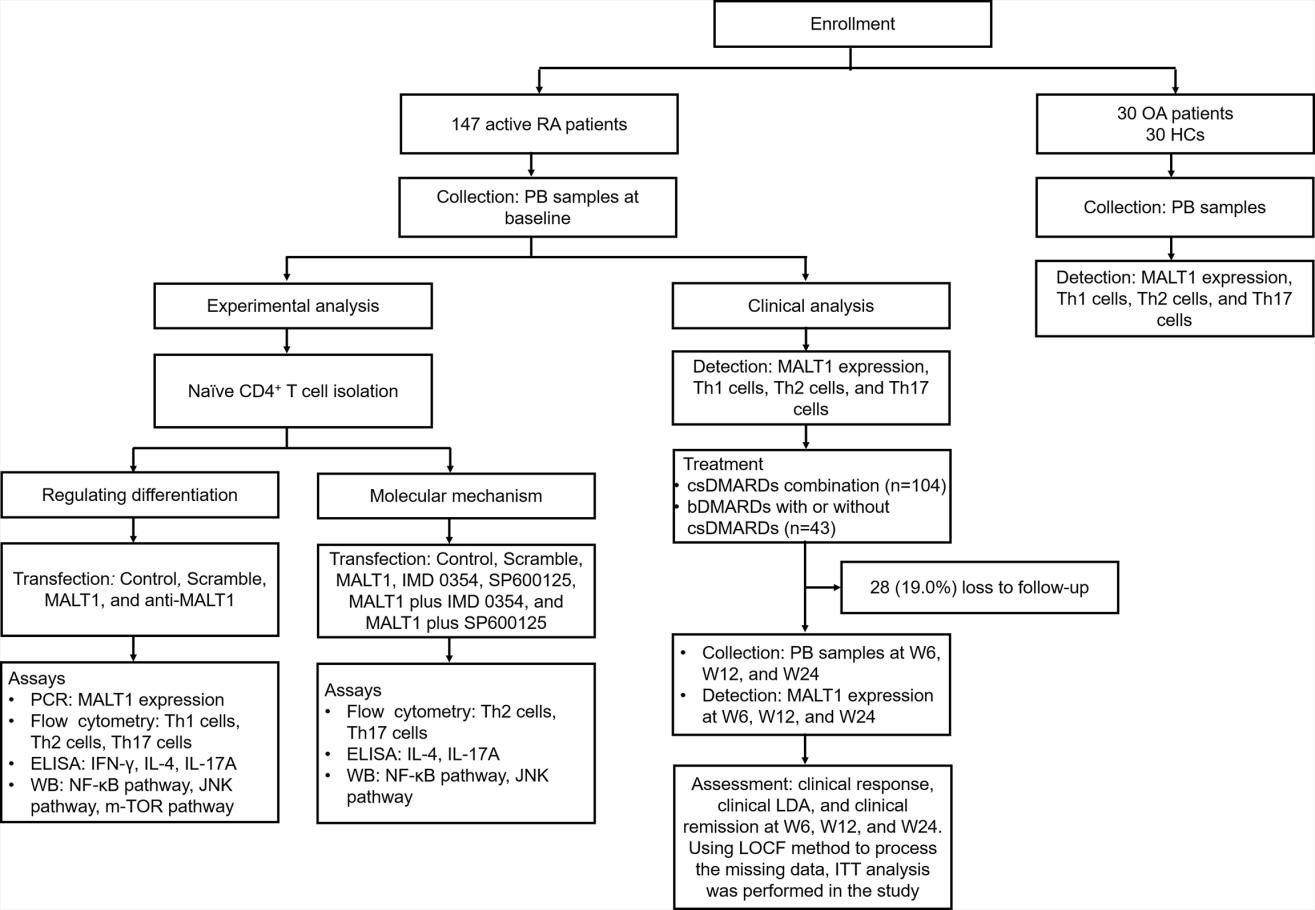
Study flow.

Clinically, 147 active RA patients were enrolled, then their PB samples were collected at baseline to detect MALT1 expression, Th1 cells, Th2 cells, and Th17 cells. Their PB samples were also collected at W6, W12, and W24 to detect MALT1 expression. Besides, clinical response, LDA, and remission at W6, W12, and W24 were evaluated. To set the controls, 30 OA patients (disease controls) and 30 HCs were also enrolled, their MALT1 expression, Th1 cells, Th2 cells, and Th17 cells were measured. Experimentally, naïve CD4^+^ T cell was isolated from RA patients, followed by lentivirus transfection, IMD 0354 treatment, SP600125 treatment, and polarization assay, then Th1, Th2, Th17 cells, and their related cytokines were detected; meanwhile, NF-κB, JNK, mTOR pathways were also measured. The detailed information of the study flow is presented in **Figure 1**.

### MALT1 was overexpressed and related to Th2, Th17 cells in RA patients

A total of 147 RA patients were enrolled with the age of 56.1 ± 9.6 years. The disease duration was 4.1 ± 3.2 years, which meant the time from diagnosis to the study enrollment. All patients were previously treated, among which 93.9%, 86.4%, 89.8%, and 26.5% patients had history of NSAID, GC, csDMARDs, and bDMARDs, respectively. While, due to flared disease activity (active disease defined as DAS28>3.2), they initiated the current treatment (70.7% csDMARDs combination and 29.3% bDMARDs with/without csDMARDs). The erythrocyte sedimentation rate (ESR) was 35.4 [interquartile range (IQR): 22.9-47.7] mm/h, C-reactive protein (CRP) was 25.5 (IQR: 12.5-39.3) mg/L. Besides, the DAS28_ESR_ score was 5.1 ± 0.7. The detailed information of RA patients’ characteristics is exhibited in **Table 1**.

**Table 1:** Clinical characteristics of RA patients.

Characteristics	RA patients (N = 147)
**Demographics**	
Age (years), mean ± SD	56.1 ± 9.6
Gender, No. (%)	
Male	27 (18.4)
Female	120 (81.6)
BMI (kg/m^2^), mean ± SD	22.7 ± 2.9
**Disease characteristics**	
Disease duration (years), mean ± SD	4.1 ± 3.2
RF positive, No. (%)	
No	29 (19.7)
Yes	118 (80.3)
ACPA positive, No. (%)	
No	44 (29.9)
Yes	103 (70.1)
TJC, median (IQR)	7.0 (5.0-9.0)
SJC, median (IQR)	6.0 (4.0-9.0)
ESR (mm/h), median (IQR)	35.4 (22.9-47.7)
CRP (mg/L), median (IQR)	25.5 (12.5-39.3)
DAS28_ESR_ score, mean ± SD	5.1 ± 0.7
CDAI score, mean ± SD	27.4 ± 7.8
HAQ-DI score, mean ± SD	1.2 ± 0.3
**Treatment history**	
History of NSAID, No. (%)	138 (93.9)
History of GC, No. (%)	127 (86.4)
History of csDMARDs, No. (%)	132 (89.8)
History of bDMARDs, No. (%)	39 (26.5)
**Current treatment regimen**	
csDMARDs combination, No. (%)	104 (70.7)
MTX+LEF	24 (16.3)
MTX+HCQ	6 (4.1)
MTX+SSZ	7 (4.8)
MTX+HCQ+SSZ	57 (38.8)
MTX+LEF+HCQ	10 (6.8)
bDMARDs with/without csDMARDs, No. (%)	43 (29.3)
ETN+MTX	23 (15.6)
IFX+MTX	4 (2.7)
ADA+MTX	7 (4.8)
TCZ+MTX	7 (4.8)
TCZ alone	2 (1.4)

RA, rheumatoid arthritis; SD, standard deviation; BMI, body mass index; RF, rheumatoid factor; ACPA, anti-cyclic citrullinated peptide antibody; TJC, tender joint count; IQR, interquartile range; SJC, swollen joint count; ESR, erythrocyte sedimentation rate; CRP, C-reactive protein; DAS28_ESR_, disease activity score 28 based on erythrocyte sedimentation rate; CDAI, clinical disease activity index; HAQ-DI, health assessment questionnaire disability index; NSAID, non-steroidal anti-inflammatory drug; GC, glucocorticoid; csDMARDs, conventional synthetic disease-modifying antirheumatic drugs; bDMARDs, biological disease-modifying antirheumatic drugs; MTX, methotrexate; LEF, leflunomide; HCQ, hydroxychloroquine; SSZ, sulfasalazine; ETN, etanercept; IFX, infliximab; ADA, adalimumab; TCZ, tocilizumab.

**Figure 2:**
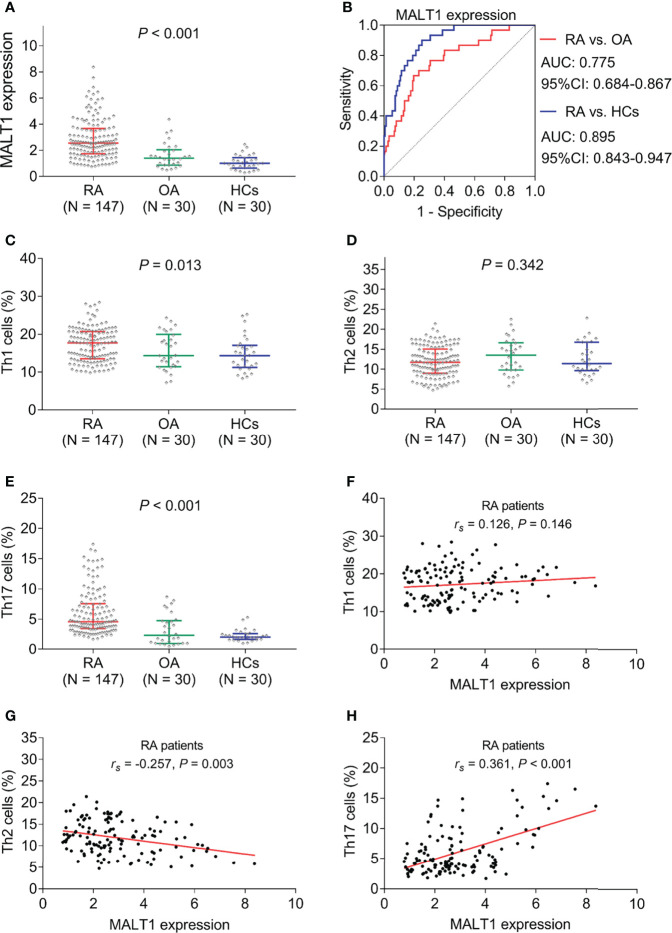
MALT1, Th1 cells, Th2 cells, and Th17 cells among subjects. MALT1 expression among RA patients, OA patients, and HCs **(A)**. ROC curve analysis of MALT1’s value in differentiating RA patients from OA patients and HCs **(B)**. Th1 cells **(C)**, Th2 cells **(D)**, and Th17 cells **(E)** among RA patients, OA patients, and HCs. Correlation of MALT1 with Th1 cells **(F)**, Th2 cells **(G)**, and Th17 cells **(H)** in RA patients.

MALT1 expression was highest in RA patients [2.550 (IQR: 1.729-3.672)], followed by OA patients [1.391 (IQR: 0.869-2.046)], then lowest in HCs [1.000 (IQR: 0.640-1.440)] (*P*<0.001, **Figure 2A**). Further ROC curve analyses observed that MALT1 was closely related to RA risk (RA *vs*. OA, AUC: 0.775) (RA *vs*. HCs, AUC: 0.895) (**Figure 2B**). Besides, MALT1 was also higher in RA patients compared to reactive arthritis patients (*P*<0.001, **Supplementary Figure 1A**), which also related to RA risk *vs*. reactive arthritis with AUC: 0.752 (**Supplementary Figure 1B**).

Th1 cells (*P*=0.013) and Th17 cells (*P*<0.001) were found to be highest in RA patients, followed by OA patients, then lowest in HCs; while Th2 (*P*=0.342) showed no difference among them (**Figures 2C–E**). Interestingly, in RA patients, MALT1 was not related to Th1 cells (*P*=0.146, **Figure 2F**), negatively correlated with Th2 cells (*P*=0.003, **Figure 2G**), positively associated with Th17 cells (*P*<0.001, **Figure 2H**).

### MALT1 was correlated with systemic inflammation and disease activity of RA

MALT1 was positively related to ESR (*P*=0.003), CRP (*P*<0.001), DAS28_ESR_ score (*P*=0.003), and CDAI score (*P*=0.030) in RA patients, but it was not correlated with other clinical features, treatment histories, and current treatment choices in RA patients (**Table 2**).

**Table 2:** Correlation of MALT1 expression with clinical characteristics in RA patients.

Characteristics	MALT1 expressionMedian (IQR)	Correlation coefficient (*r_s_*)	*P* value
Age	–	0.076	0.363
BMI	–	0.115	0.167
Disease duration	–	0.113	0.173
TJC	–	0.137	0.098
SJC	–	0.110	0.183
ESR	–	0.240	**0.003**
CRP	–	0.298	**<0.001**
DAS28_ESR_ score	–	0.242	**0.003**
CDAI score	–	0.179	**0.030**
HAQ-DI score	–	0.128	0.121
Gender		–	0.555
Male	2.604 (2.007-4.308)		
Female	2.476 (1.716-3.654)		
RF positive		–	0.112
No	2.676 (1.912-5.433)		
Yes	2.476 (1.724-3.510)		
ACPA positive		–	0.060
No	2.679 (2.179-3.871)		
Yes	2.411 (1.623-3.500)		
History of NSAID		–	0.263
No	2.086 (1.400-3.598)		
Yes	2.566 (1.789-3.672)		
History of GC		–	0.678
No	2.466 (1.648-3.887)		
Yes	2.557 (1.729-3.672)		
History of csDMARDs		–	0.267
No	2.086 (1.552-2.641)		
Yes	2.566 (1.773-3.673)		
History of bDMARDs		–	0.732
No	2.513 (1.807-3.593)		
Yes	2.602 (1.576-3.763)		
Current treatment: csDMARDs combination		–	0.116
No	2.527 (2.061-4.137)		
Yes	2.554 (1.616-3.531)		
Current treatment: bDMARDs with/without csDMARDs		–	0.116
No	2.554 (1.616-3.531)		
Yes	2.527 (2.061-4.137)		

MALT1, mucosa-associated lymphoid tissue lymphoma translocation protein 1; RA, rheumatoid arthritis; IQR, interquartile range; BMI, body mass index; TJC, tender joint count; SJC, swollen joint count; ESR, erythrocyte sedimentation rate; CRP, C-reactive protein; DAS28_ESR_, disease activity score 28 based on erythrocyte sedimentation rate; CDAI, clinical disease activity index; HAQ-DI, health assessment questionnaire disability index; RF, rheumatoid factor; ACPA, anti-cyclic citrullinated peptide antibody; NSAID, non-steroidal anti-inflammatory drug; GC, glucocorticoid; csDMARDs, conventional synthetic disease-modifying antirheumatic drugs; bDMARDs, biological disease-modifying antirheumatic drugs. Bold values meant statistical significance (P < 0.05).

Subsequently, in order to investigate the treatment regimen on outcomes, MALT1 and Th cells levels, we divide the various regimens into 4 kinds for analysis: (1) double csDMARDs combination; (2) triple csDMARDs combination; (3) TNFi+MTX; (4) TCZ ± MTX. Then it was observed that in RA patients (**Table 3**): Firstly, Response rate showed a different trend but no statistical significance (*P*=0.083) among double csDMARDs combination (37.8%), triple csDMARDs combination (59.7%), TNFi+MTX (64.7%), and TCZ ± MTX (44.4%), respectively; Secondly, LDA rate (*P*=0.374) and remission rate (*P*=0.720) were not different among these four kinds of treatments; Thirdly, MALT1 (*P*=0.317), Th1 cells (*P*=0.292), and Th17 cells (*P*=0.905) were not different by these four kinds of treatments; Fourthly, Th2 cells (*P*=0.042) were varied by these four kinds of treatments.

**Table 3:** Correlation of current treatment regimen kinds with outcomes, MALT1 level, and Th cells level in RA patients.

Parameters	Double csDMARDs combination	Triple csDMARDs combination	TNFi+MTX	TCZ ± MTX	*P* value
Response rate	37.8%	59.7%	64.7%	44.4%	0.083
LDA rate	29.7%	40.3%	50.0%	44.4%	0.374
Remission rate	24.3%	26.9%	35.3%	22.2%	0.720
MALT1, median (IQR)	2.575 (1.552-3.780)	2.601 (1.698-3.754)	2.540 (2.179-4.280)	2.527 (1.788-5.242)	0.317
Th1 cells, median (IQR)	14.3% (12.7%-20.7%)	17.5% (13.8%-20.7%)	18.5% (1 4.4%-21.0%)	14.5% (11.2%-20.5%)	0.292
Th2 cells, median (IQR)	12.6% (9.7%-16.4%)	12.3% (9.4%-16.1%)	10.5% (7.6%-12.2%)	12.0% (9.0%-13.6%)	0.042
Th17 cells, median (IQR)	4.6% (3.2%-8.4%)	5.0% (3.7%-7.3%)	4.2% (3.0%-9.8%)	3.9% (3.1%-9.8%)	0.905

MALT1, mucosa-associated lymphoid tissue lymphoma translocation protein 1; Th, T helper; RA, rheumatoid arthritis; csDMARDs, conventional synthetic disease-modifying antirheumatic drugs; TNFi, tumor necrosis factor inhibitor; TCZ, tocilizumab; MTX, methotrexate; LDA, low disease activity.

### MALT1 longitudinal reduction was related to RA treatment outcome

**Figure 3:**
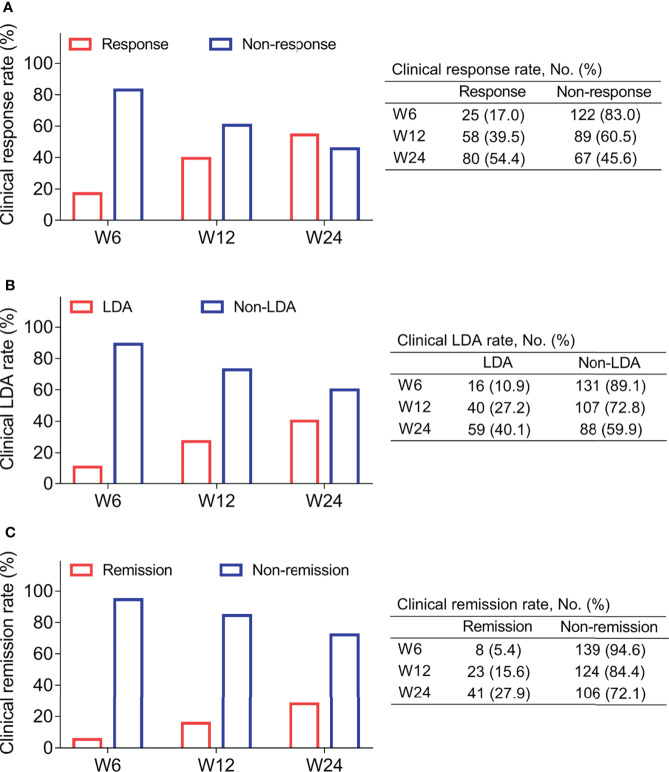
Treatment outcome. Clinical response rate **(A)**, clinical LDA rate **(B)**, and clinical remission rate **(C)** at W6, W12, and 24 in RA patients.

After treatment, 17.0%, 39.5%, and 54.4% RA patients realized clinical response at W6, W12, and W24, respectively (**Figure 3A**). At the same time, 10.9%, 27.2%, and 40.1% of RA patients achieved clinical LDA at W6, W12, and W24, respectively (**Figure 3B**). Besides, 5.4%, 15.6%, and 27.9% of RA patients reached clinical remission at W6, W12, and W24, respectively (**Figure 3C**).

**Figure 4:**
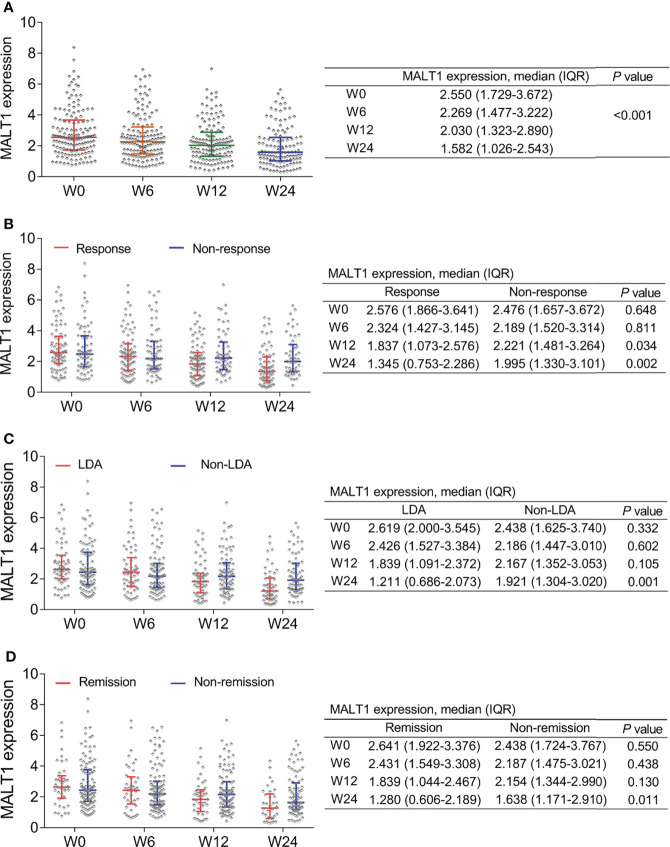
MALT1’s change during treatment and outcome. Longitudinal change of MALT1 expression during treatment in RA patients **(A)**. Comparison of MALT1 expression at W0, W6, W12, and W24 between response patients and non-response patients **(B)**, between LDA patients and non-LDA patients **(C)**, between remission patients and non-remission patients **(D)**.

MALT1 decreased gradually from baseline/W0 [2.550 (IQR: 1.729-3.672)] to W24 [1.582 (IQR: 1.026-2.543)] (*P*<0.001, **Figure 4A**). Notably, MALT1 at W12 (*P*=0.034) and W24 (*P*=0.002) was lower in response patients compared to non-response ones (**Figure 4B**). Furthermore, MALT1 at W24 was also lower in LDA patients compared to non-LDA ones (*P*=0.001, **Figure 4C**), in remission patients compared to non-remission ones (*P*=0.011, **Figure 4D**). The findings indicated MALT1 longitude decrement related to the better treatment outcomes. In addition, it was also observed that baseline MALT1 level was not differed between response patients and non-response patients (*P*=0.648), between LDA patients and non-LDA patients (*P*=0.332), between remission patients and non-remission patients (*P*=0.550) (**Figures 4B–D**). These indicated baseline MALT1 could not predict treatment outcomes

In addition, when clinical remission and LDA was assessed by CDAI, it was also observed that MALT1 at W24 was lower in LDA (by CDAI) patients compared to non-LDA (by CDAI) patients (*P*=0.003, **Supplementary Figure 2A**), as well as in remission (by CDAI) patients compared to non-remission (by CDAI) patients (*P*=0.040, **Supplementary Figure 2B**).

### MALT1 promoted Th17 differentiation but repressed Th2 differentiation in RA

**Figure 5:**
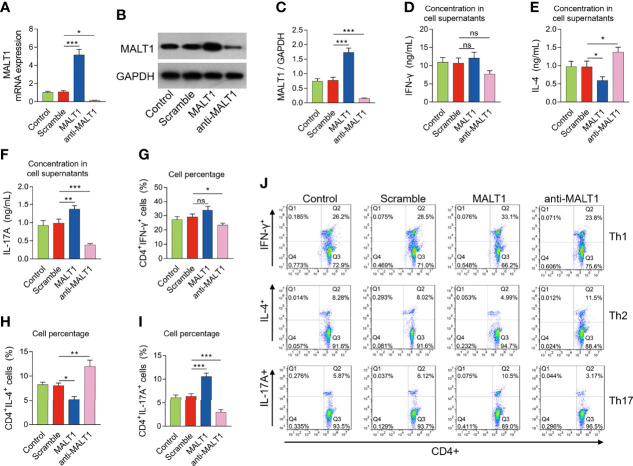
Effect of MALT1 on Th1/2/17 cell differentiation in RA. MALT1 mRNA expression **(A)**, MALT1 protein expression **(B, C)** after lentivirus transfection. IFN-γ level **(D)**, IL-4 level **(E)**, IL-17A level **(F)** in cell supernatants, percentage of CD4^+^ IFN-γ^+^ cells **(G)**, CD4^+^ IL-4^+^ cells **(H)**, CD4^+^ IL-17A^+^ cells **(I)** after lentivirus transfection. Examples of flow cytometry image after lentivirus transfection **(J)**. * meant P<0.05, ** meant P<0.01, *** meant P<0.001, ns meant not significant.

After lentivirus transfection, MALT1 expression was modified in RA CD4^+^ T cells accordingly (**Figures 5A–C**). Then MALT1 overexpression did not affect the IFN-γ level (*P*>0.05), reduced the IL-4 level (*P*<0.05), but elevated the IL-17A level (*P*<0.01) after polarization assay (**Figures 5D–F**). Meanwhile, MALT1 overexpression did not influence the CD4^+^ IFN-γ^+^ cells (*P*>0.05), decreased the CD4^+^ IL-4^+^ cells (*P*<0.05), but increased the CD4^+^ IL-17A^+^ cells (*P*<0.001) after polarization assay (**Figures 5G–J**). On the contrary, MALT1 knockdown raised the IL-4 level (*P*<0.05) and CD4^+^ IL-4^+^ cells (*P*<0.01), but reduced the IL-17A level (*P*<0.001), CD4^+^ IFN-γ^+^ cells (*P*<0.05), and CD4^+^ IL-17A^+^ cells (*P*<0.001) after polarization assay (**Figures 5D–J**).

Meanwhile, it was observed that MALT1 mRNA expression (**Supplementary Figure 3A**) and protein expression (**Supplementary Figures 3B, C**) were not changed in Th1 polarized cells, Th2 polarized cells, or Th17 polarized cells without lentiviral infection compared to control cells.

### MALT1 activated NF-κB and JNK pathways in RA CD4^+^ T cells

**Figure 6:**
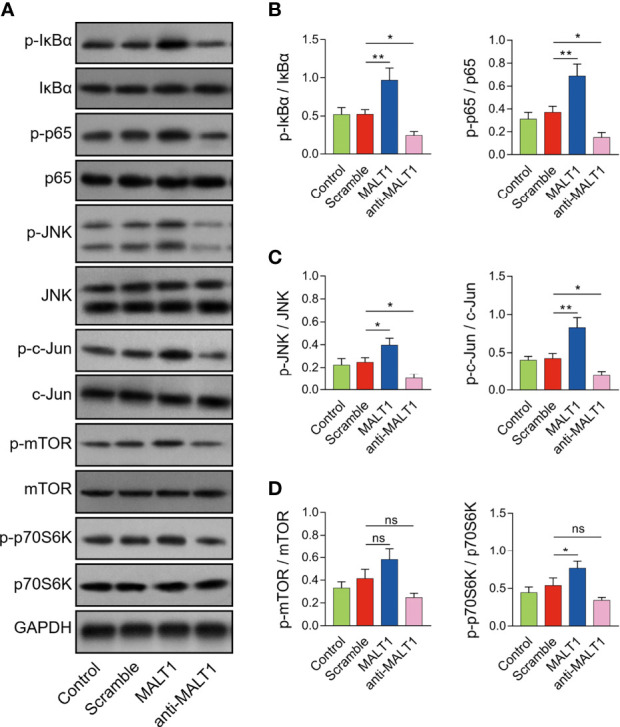
Effect of MALT1 on NF-κB, JNK, and mTOR pathways in RA CD4^+^ T cells. Examples of corresponding western blot images after lentivirus transfection **(A)**. p-IκBα and p-p65 **(B)**, p-JNK and p-c-Jun **(C)**, p-mTOR and p-p70S6K **(D)** protein expressions after lentivirus transfection. * meant P<0.05, ** meant P<0.01, ns meant not significant.

MALT1 overexpression increased p-IκBα (*P*<0.01) and p-p65 expressions (*P*<0.01) (**Figures 6A, B**), also elevated p-JNK (*P*<0.05) and p-c-Jun expressions (*P*<0.05) (**Figures 6A, C**), but less affected p-mTOR or p-p70S6K expressions (**Figures 6A, D**) in RA CD4^+^ T cells. On the contrary, MALT1 knockdown had an opposite effect on these protein expressions as its overexpression did (**Figures 6A–D**).

### MALT1 regulated Th2 and Th17 differentiation *via* NF-κB and JNK pathways in RA

**Figure 7:**
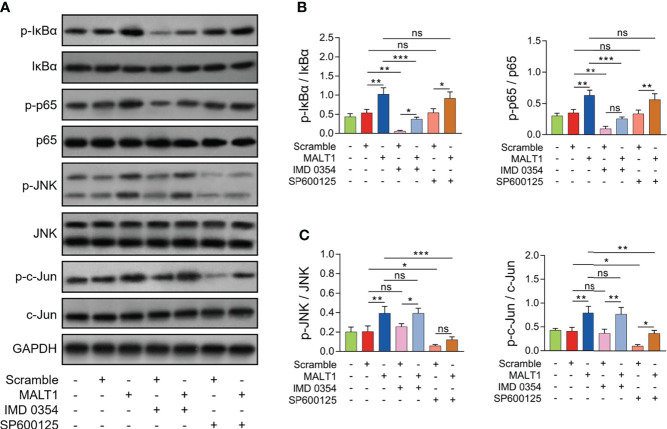
Effect of IMD 0354 and SP600125 treatment on NF-κB and JNK pathways in RA CD4^+^ T cells. Examples of corresponding western blot images after lentivirus transfection, IMD 0354 treatment, SP600125 treatment, alone or in combination **(A)**. p-IκBα and p-p65 **(B)**, p-JNK and p-c-Jun **(C)** protein expressions after lentivirus transfection, IMD 0354 treatment, SP600125 treatment, alone or in combination. * meant P<0.05, ** meant P<0.01, *** meant P<0.001. ns meant not significant.

Generally, the effect of IMD 0354 (NF-κB antagonist) on inactivating the NF-κB pathway was realized in RA CD4^+^ T cells (**Figures 7A, B**). Meanwhile, the effect of SP600125 (JNK antagonist) on inactivating the JNK pathway was also realized in RA CD4^+^ T cells (**Figures 7A, C**).

**Figure 8:**
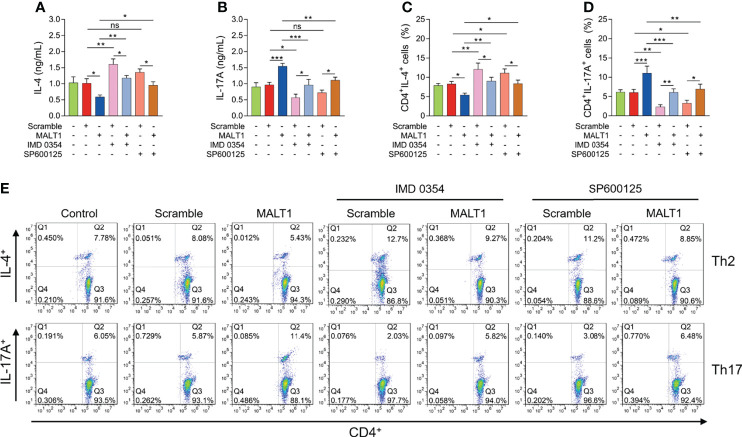
Effect of IMD 0354 and SP600125 treatment on Th1/2/17 cell differentiation in RA. IL-4 level **(A)**, IL-17A level **(B)**, CD4^+^ IL-4^+^ cells **(C)**, CD4^+^ IL-17A^+^ cells **(D)** after lentivirus transfection, IMD 0354 treatment, SP600125 treatment, alone or in combination. Examples of flow cytometry images after lentivirus transfection, IMD 0354 treatment, SP600125 treatment, alone or in combination **(E)**. * meant P<0.05, ** meant P<0.01, *** meant P<0.001. ns meant not significant.

IMD 0354 treatment increased IL-4 level (*P*<0.01) but decreased IL-17A level (*P*<0.01) (**Figures 8A, B**). At the same time, IMD 0354 treatment elevated CD4^+^ IL-4^+^ cells (*P*<0.01) but reduced CD4^+^ IL-17A^+^ cells (*P*<0.05) (**Figures 8C–E**). More importantly, IMD 0354 treatment attenuated the effect of MALT1 on the above indexes (**Figures 8A–E**).

In addition, SP600125 treatment did not regulate IL-4 level (*P*>0.05) or IL-17A level (*P*>0.05) (**Figures 8A, B**). However, SP600125 treatment elevated CD4^+^ IL-4^+^ cells (*P*<0.05) but reduced CD4^+^ IL-17A^+^ cells (*P*<0.05) (**Figures 8C–E**). Besides, the SP600125 treatment weakened the effect of MALT1 on the above indexes (**Figures 8A–E**).

**Figure 9:**
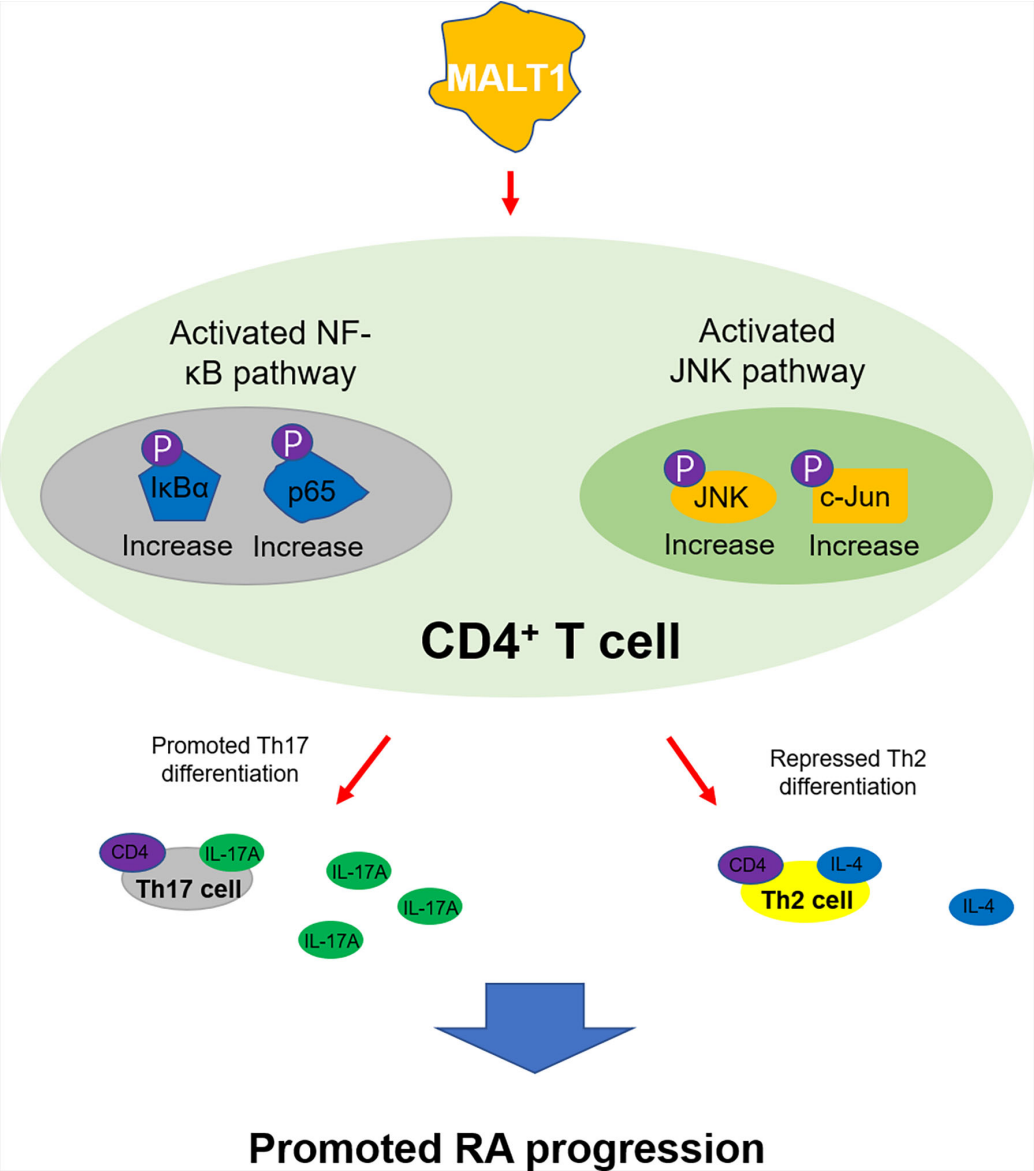
The mechanism of MALT1 on regulating RA Th2 and Th17 differentiation.

Comminating all the findings into a model (**Figure 9**), It was shown that MALT1 activated NF-κB and JNK pathways in CD4^+^ T cells, promoted their differentiation into Th17 cells while repressed their differentiation into Th2 cells, then to accelerated RA progression.

## Discussion

MALT1 is a recently identified multi-functional factor involved in the regulation of immune cells and inflammation (9–12), resulting from which MALT1 is dysregulated and correlates with disease activity in immune and inflammatory diseases (14–16, 20). For instance, MALT1 is upregulated in Crohn’s disease patients and ulcerative colitis patients compared to HCs, and it relates to elevated CRP, ESR, Crohn’s disease activity index score, or mayo score in the active patients (14). Besides, MALT1 is also reported to overexpress in RA patients compared to HCs (20); however, the previous study lacks the enrollment of disease controls, evaluation of diagnostic value as well as multiple disease features, and has a relatively small sample size (20). In our current study, it was observed that MALT1 was increased in RA patients versus OA patients (disease controls) and HCs, which shows a close relation to RA risk by ROC curve analyses; meanwhile, it correlated with higher CRP, ESR, and DAS28 score in RA patients. The possible explanations are: (1) MALT1 activates NF-κB and MAPK pathways to regulate immune response and inflammation (9–12), which increase the risk and activity of RA; (2) MALT1 regulates T cell functions to directly affects RA development (18).

Since MALT1 could reflect disease risk and activity of several autoimmune diseases (14–16), it’s interesting to explore whether it could serve as a biomarker for disease monitoring. A previous study observes that MALT1 gradually decreases during treatment in active IBD patients, and its decrement links with treatment response to some extent (14). In the current study, MALT1 was observed to reduce longitudinally during treatment, whose reduction correlated with RA treatment outcome (treatment response, low disease activity, or disease remission), which was in line with the previous study in IBD patients (14). The explanations are: (1) during treatment, the inflammation of RA is attenuated, then MALT1 closely relates to RA inflammation, so MALT1 is decreased together with inflammation (12, 14, 20); (2) bDMARDs or csDMARDs treatment may regulate multiple immune cells to affect MALT1 level in patients (29, 30). The above data indicated the value of MALT1 measurement as a marker for disease monitoring of RA. MALT1 may better reflect disease activity and treatment outcomes using a robust tool such as CDAI; meanwhile, it closely relates to Th cell dysregulation in RA patients.

MALT1 is recently discovered to regulate T-cell receptors and modify Th cells (31). Then another study further finds MALT1 inhibition represses Th1, Th17, Th1/Th17 effector response, as well as retards T-cell dependent B-cell response (32). Besides, knocking down MALT1 ameliorates colitis *via* blocking Th17 and Th1/17 cells activation (33). Apart from the above findings, clinically, MALT1 also discloses a positive linkage with Th1, Th17, or their secreted cytokines in several immune or inflammatory diseases such as IBD, acute ischemic stroke, etc (14, 34). Besides, a recent study observes an inter-correlation among MALT1, Th1 cells, and Th17 cells in RA patients; meanwhile, they are dysregulated and relates to disease activity as well (20). Inspired by the reports of MALT1’s relationship with Th cells in several studies, and the implication of Th cells in RA etiology and treatment, the effect of MALT1 on Th1/2/17 cell differentiation was subsequently investigated in this study. It was then observed that MALT1 positively correlated with Th17 cells but negatively associated with Th2 cells in RA patients; meanwhile, MALT1 promoted Th17 cell differentiation, inhibited Th2 cell differentiation, less affected Th1 cell differentiation in RA CD4^+^ T cells. The possible explanation is that MALT1 regulates NF-κB and JNK pathways in RA CD4^+^ T cells to improve Th17 cell differentiation but to repress Th2 cell differentiation, which is validated by our subsequent experiments.

NF-κB and JNK pathways are well-known to implicate in RA pathology (35); meanwhile, they could also regulate CD4^+^ T cell differentiation in several immune and inflammatory diseases (36, 37). For instance, the NF-κB pathway activates Th17 differentiation to involve in psoriasis development (36); the JNK pathway promotes CD4^+^ T cell infiltration in Sjogren’s syndrome (37). In our current study, whether MALT1 regulated Th2 and Th17 differentiation *via* NF-κB and JNK pathways were further explored by adding IMD 0354 or SP600125 into MALT1 treated RA CD4^+^ T cells. Then it was observed that both IMD 0354 and SP600125 promoted Th2 cell differentiation but reduced Th17 cell differentiation in RA CD4^+^ T cells; notably, they attenuated MALT1’s effect on Th2 and Th17 differentiation. These data indicated MALT1 modified Th2 and Th17 differentiation through NF-κB and JNK pathways in RA.

Some limitations could be mentioned in this study. Firstly, as a potential disease monitoring marker, the correlation of MALT1 with flare risk in LDA/remission patients could be further evaluated. Secondly, MALT1’s effect on other immune cells (such as B cells, NK cells, Tregs) apart from Th cells in RA was not conducted in this study, which could be investigated in the future study. Thirdly, the study found MALT1 regulated NF-κB and JNK pathways, the latter two were also essential in osteoclast differentiation, therefore, the effect of MALT1 on bone damage of RA could be explored in the future.

In conclusion, MALT1 regulates Th2 and Th17 differentiation *via* NF-κB and JNK pathways, as well as correlates with disease activity and treatment outcome in RA.

## Data Availability Statement

The original contributions presented in the study are included in the article/**supplementary material**. Further inquiries can be directed to the corresponding author/s.

## Ethics Statement

This study was reviewed and approved by Ethics Committee of Wuxi 9th People's Hospital Affiliated to Soochow University. The patients/participants provided their written informed consent to participate in this study.

## Author Contributions

RD and JM contributed to the conception and design of the study. QW, YW and QL performed the experiment. YC, RM and FJ contributed to the data acquisition. JZ, KH and YF contributed to the analysis and interpretation of data. RL, HL and DZ contributed to drafting/revising of article. All authors read and approved the final manuscript. All authors contributed to the article and approved the submitted version.

## Funding

This study was supported by Scientific Research Project of Wuxi Science and Technology Bureau (Y20212054), Innovation Project (Ph.D) of Wuxi 9th People’ s Hospital Affiliated to Soochow University (YB202101) and Innovation Project (Ph.D) of Wuxi 9th People’ s Hospital Affiliated to Soochow University (YB202110).

## Conflict of Interest

Authors JZ, KH, RL, YF, DZ and RD were employed by Shanghai QeeJen Bio-tech Institution.

The remaining authors declare that the research was conducted in the absence of any commercial or financial relationships that could be construed as a potential conflict of interest.

## Publisher’s Note

All claims expressed in this article are solely those of the authors and do not necessarily represent those of their affiliated organizations, or those of the publisher, the editors and the reviewers. Any product that may be evaluated in this article, or claim that may be made by its manufacturer, is not guaranteed or endorsed by the publisher.
