# Supplementary material for: MALT1 regulates Th2 and Th17 differentiation via NF-κB and JNK pathways, as well as correlates with disease activity and treatment outcome in rheumatoid arthritis
Source: Front Immunol. 2022 Jul 28;13:913830. doi: 10.3389/fimmu.2022.913830 (PMC9367691; doi:10.3389/fimmu.2022.913830)
Supplement: Supplementary file 1 [file DataSheet_1.docx]

***Supplementary Material***

**Supplementary table 1.** Antibodies information of western blot assays.

| Antibody | Company | Dilution |
| --- | --- | --- |
| Anti-MALT1 antibody | Santa Cruz (USA) | 1:200 |
| Anti-p-IκBα antibody | Abcam (UK) | 1:10000 |
| Anti-IκBα antibody | Abcam (UK) | 1:10000 |
| Anti-p-p65 antibody | Abcam (UK) | 1:1000 |
| Anti-p65 antibody | Abcam (UK) | 1:3000 |
| Anti-p-JNK antibody | Santa Cruz (USA) | 1:200 |
| Anti-JNK antibody | Santa Cruz (USA) | 1:200 |
| Anti-p-c-Jun antibody | Santa Cruz (USA) | 1:200 |
| Anti-c-Jun antibody | Santa Cruz (USA) | 1:200 |
| Anti-p-mTOR antibody | CST (USA) | 1:1000 |
| Anti-mTOR antibody | CST (USA) | 1:1000 |
| Anti-p-p70S6K antibody | CST (USA) | 1:1000 |
| Anti-p70S6K antibody | CST (USA) | 1:1000 |
| Anti-GAPDH antibody | Abcam (UK) | 1:3000 |
| Goat Anti-Rabbit IgG HRP | Abcam (UK) | 1:5000 |
| Goat Anti-Mouse IgG HRP | Abcam (UK) | 1:5000 |

**
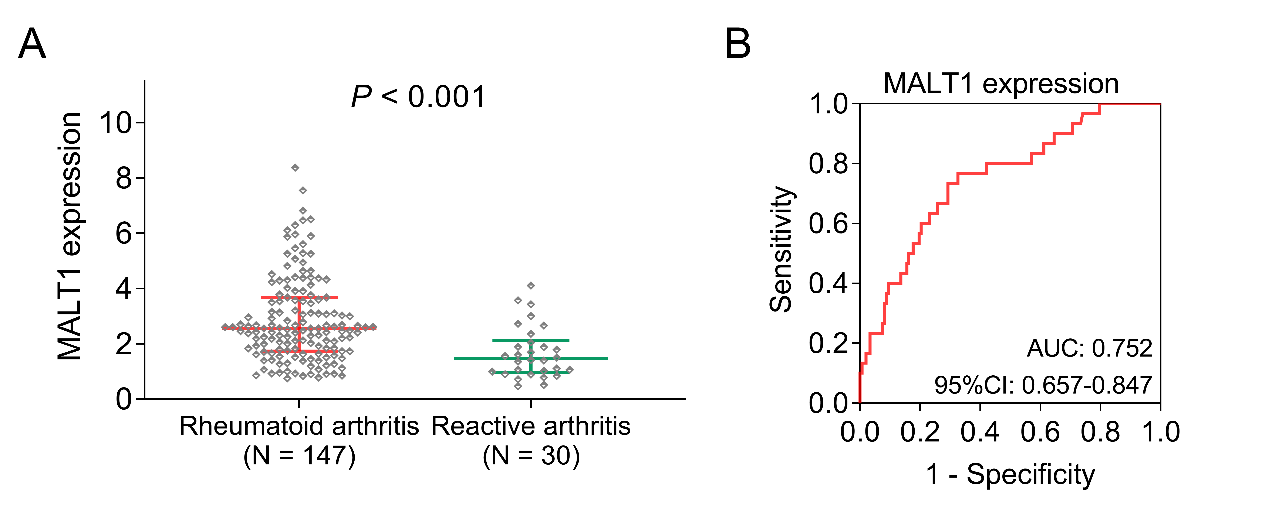
**

**Supplementary figure 1.** MALT1 between rheumatoid arthritis patients and reactive arthritis patients. Comparison of MALT1 level between rheumatoid arthritis patients and reactive arthritis patients (A). ROC curve analysis of MALT1’s value in differentiating rheumatoid arthritis patients from reactive arthritis patients (B).


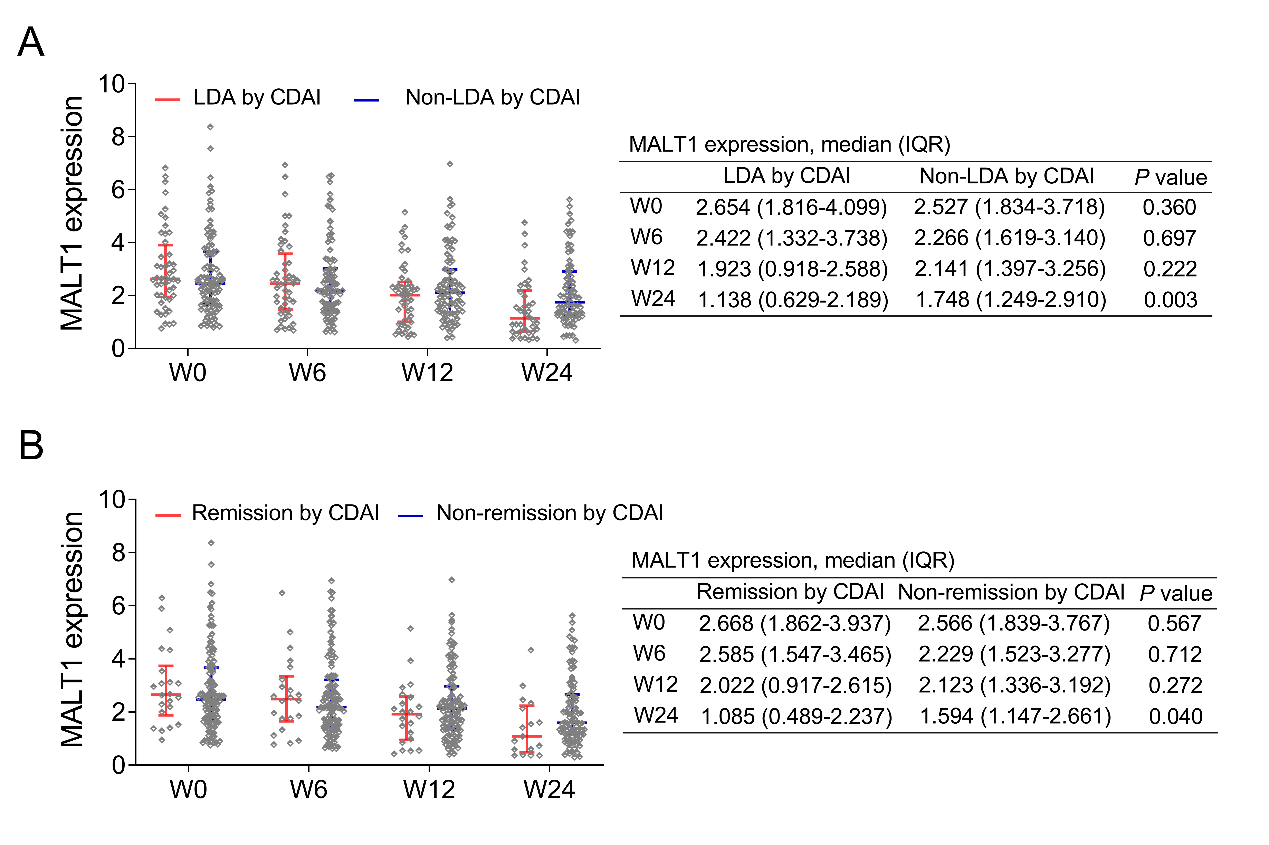


**Supplementary figure 2.** Correlation of MALT1’s change with LDA and remission by CDAI. Comparison of MALT1 expression at W0, W6, W12, and W24 between between LDA patients and non-LDA patients assessed by CDAI score (A), between remission patients and non-remission patients assessed by CDAI score (B).


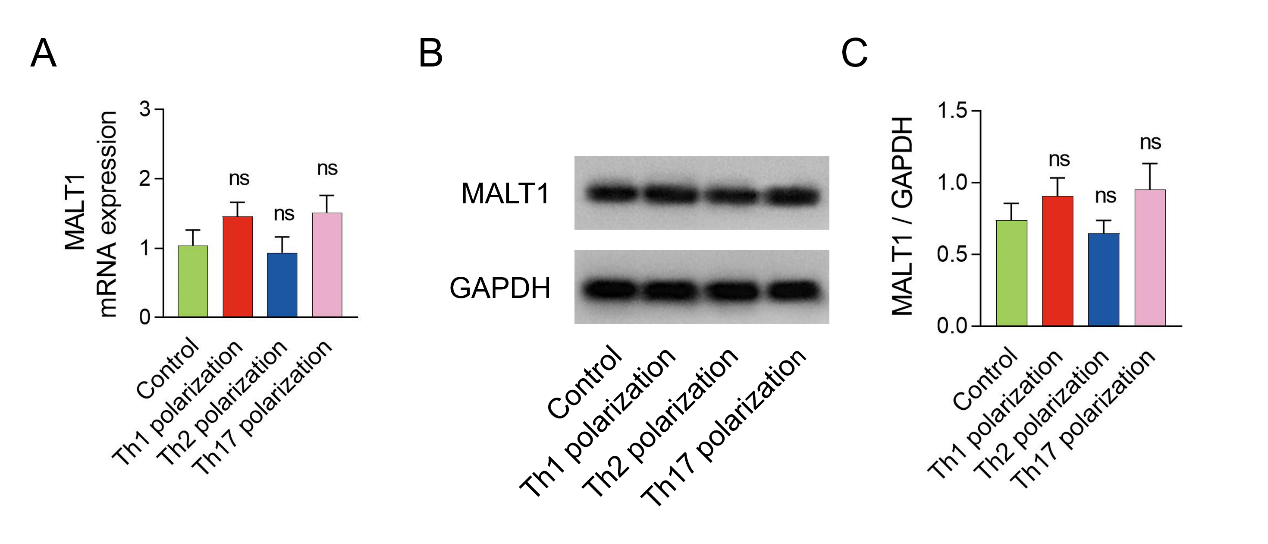


**Supplementary figure 3.** MALT1 expression after Th polarization. MALT1 mRNA expression (A), western blot image examples of MALT1 (B), quantification of MATL1 protein expression (C) in Th1 polarized cells, Th2 polarized cells, and Th17 polarized cells.
